# Study Protocol and Baseline Cardiometabolic Characterization of the RIO-Study (Response to an Intervention with Omega-3): A Randomized, Double-Blind, Placebo-Controlled Crossover Trial on Lipid and Inflammatory Profiles in Overweight and Obese Adults with Hypertriglyceridemia in Valdivia, Chile

**DOI:** 10.3390/nu17213397

**Published:** 2025-10-29

**Authors:** Josefina Enríquez, Consuelo Quezada, Jessica Molina, Matías Sáez, Iarela Mitre, Camila Moreira, Feren Sandoval, Rodrigo Maldonado, Montserrat Fitó, Sebastián Zagmutt, Catalina Ramírez-Contreras, Eneko Ganuza, Álvaro Hernáez, Sergio Martínez-Huenchullán, Viviana Sandoval

**Affiliations:** 1 Carrera de Nutrición y Dietética, Facultad de Ciencias de la Rehabilitación y Calidad de Vida, Universidad San Sebastián, Valdivia 5090000, Chile; josefina.enri.sch@gmail.com (J.E.); consuelo.quezada99@gmail.com (C.Q.); jessica.molina@uss.cl (J.M.); msaezg5@correo.uss.cl (M.S.); imitrec@correo.uss.cl (I.M.); cmoreiraa1@correo.uss.cl (C.M.); fsandovalj@correo.uss.cl (F.S.); 2 Departamento de Ciencias Básicas, Facultad de Ciencias, Universidad San Sebastián, Valdivia 5090000, Chile; rodrigo.maldonado@uss.cl; 3 Hospital del Mar Research Institute (HMRIB), 08003 Barcelona, Spain; mfito@researchmar.net (M.F.); alvarohc1@blanquerna.url.edu (Á.H.); 4 CIBER de Fisiopatología de la Obesidad y Nutrición (CIBEROBN), Instituto de Salud Carlos III, 28029 Madrid, Spain; 5 Department of Biomedical Sciences, Faculty of Medicine and Health Sciences, Universitat Internacional de Catalunya, 08195 Sant Cugat del Vallès, Spain; szagmutt@uic.es; 6 Departamento de Nutrición y Salud Pública, Facultad de Ciencias de la Salud y los Alimentos, Universidad del Bío-Bío, Chillán 3780000, Chile; ctramirez@ubiobio.cl; 7 Qualitas Health Inc., iwi life, Houston, TX 77056, USA; eganuza@iwilife.com; 8 Blanquerna School of Health Sciences, University Ramon Llull, 08025 Barcelona, Spain; 9 CIBER de Enfermedades Cardiovasculares (CIBERCV), Instituto de Salud Carlos III, 28029 Madrid, Spain; 10 Carrera de Kinesiología, Facultad de Ciencias de la Rehabilitación y Calidad de Vida, Universidad San Sebastián, Valdivia 5090000, Chile; 11 Centro de Biología Celular y Biomedicina (CEBICEM), Facultad de Ciencias, Universidad San Sebastián, Santiago 8580000, Chile

**Keywords:** cardiometabolic risk, dyslipidemia, inflammation, omega-3 fatty acids, overweight

## Abstract

Background: Cardiovascular diseases (CVDs) are the leading cause of morbidity and mortality worldwide, with metabolic syndrome and its risk factors contributing substantially to cases in Latin America. In southern Chile, obesity, dyslipidemia, and sedentary behavior are highly prevalent, yet comprehensive baseline data on these factors are scarce. Establishing regional cardiometabolic profiles is crucial to inform prevention strategies. Objective: To describe the RIO-Study protocol and characterize the baseline cardiometabolic profile of adults from Valdivia, southern Chile. Methods: The RIO-Study is a randomized, double-blind, placebo-controlled, crossover clinical trial evaluating the effects of nutritional doses of seaweed-derived omega-3 fatty acids on lipid metabolism, inflammation, and molecular lipid regulators in adults with overweight/obesity. The protocol includes a standardized high-fat breakfast challenge and repeated postprandial blood sampling to assess dynamic lipid responses. Screening procedures comprised blood pressure measurement, fasting blood sampling, body composition by bioelectrical impedance, and health and lifestyle questionnaires. Results: Among screened participants, 91% presented overweight/obesity and 55% presented central adiposity, exceeding regional cardiometabolic risk thresholds (waist circumference ≥ 90 cm in men, ≥80 cm in women). Men exhibited higher waist circumference (100 ± 10.8 vs. 91.6 ± 11.9 cm), waist-to-hip ratio (0.99 ± 0.08 vs. 0.92 ± 0.07), systolic blood pressure (130 ± 12.0 vs. 122 ± 13.4 mmHg), triglycerides (168 ± 84.7 vs. 122 ± 64.9 mg/dL), VLDL-C (33.7 ± 17.2 vs. 24.4 ± 13.0 mg/dL), and sedentary time (8.1 ± 2.3 vs. 6.8 ± 2.3 h/day). Women had greater total body fat (39.7 ± 4.75% vs. 31.1 ± 5.30%), higher HDL-C (56.6 ± 13.3 vs. 46.9 ± 9.39 mg/dL), and more often had normal weight (13% vs. 0%). Conclusions: The RIO-Study provides novel insights into cardiometabolic risk and will elucidate the effects of nutritional omega-3 supplementation in a high-risk Chilean population.

## 1. Introduction

Cardiovascular diseases (CVDs) represent a global health crisis, claiming approximately 17.9 million lives annually through a complex interplay of behavioral, metabolic, and genetic risk factors [1]. At the center of this burden is metabolic syndrome (MetS), a constellation of conditions including central obesity, dyslipidemia, hypertension, and hyperglycemia that significantly increases cardiovascular risk [2]. Among these, elevated fasting or postprandial triglyceride levels have emerged as independent predictors of CVD events, making them a critical therapeutic target [3]. The Latin American region, particularly Chile, exemplifies the global shift toward higher cardiometabolic risk. The country has experienced a dramatic increase in overweight and obesity, currently affecting 78.8% of adults (≥15 years), paralleled by increasing rates of central adiposity and MetS [4]. This epidemic is explained by unhealthy sedentary behaviors, with widespread failure to meet established “24-h movement guidelines” which encompass adequate physical activity, limited sedentary time, and sufficient sleep. Non-adherence to these guidelines correlates strongly with high risks of obesity, hypertension, dyslipidemia, type 2 diabetes, MetS, and long-term cardiovascular complications [5]. However, comprehensive Latin American data characterizing cardiometabolic profiles remains limited, particularly in southern Chile [6].

Omega-3 polyunsaturated fatty acids (PUFAs), notably eicosapentaenoic acid (EPA) and docosahexaenoic acid (DHA), have attracted considerable attention for their well-documented lipid-lowering and anti-inflammatory properties [7,8,9], establishing them as promising interventions for cardiometabolic risk reduction. Nevertheless, the therapeutic efficacy of omega-3 supplementation exhibits substantial interindividual variation, influenced by baseline metabolic status, dietary patterns, genetic architecture, and lifestyle factors [10,11]. While omega-3 research has flourished in high-income countries, intervention studies targeting Latin American populations remain sparse [12]. The RIO-Study trial was designed to address this knowledge gap.

Therefore, this work aims to (1) describe the design and methodology of the RIO-Study and (2) characterize the baseline cardiometabolic profile of adults from Valdivia, Chile, assessed during the screening phase.

## 2. Materials and Methods

### 2.1. Trial Design

**Figure 1:**
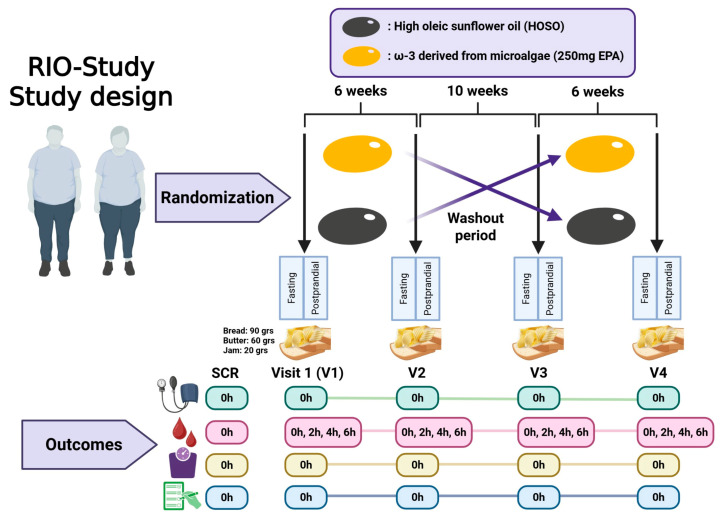
Design of a randomized crossover clinical trial with a washout period. Participants are randomly assigned to one of two interventions (Omega-3 or HOSO) and are assessed before and after the intervention. Following a washout phase, the interventions are crossed over. Outcomes are measured at different time points (0, 2, 4, and 6 h) across four visits (V1–V4) to evaluate the effects of each intervention. Figure created with BioRender.com.

The RIO-Study (Response to an Intervention with Omega-3 Fatty Acids on Lipid and Inflammatory Profiles in Overweight and Obese Individuals with Hypertriglyceridemia in Valdivia, Chile) is a randomized, double-blind, placebo-controlled, two-period, two-sequence crossover trial. Each participant completed two supplementation phases of six weeks, separated by a 10-week washout period to minimize carryover effects (Figure 1). Recruitment was conducted between July 2024 and June 2025 at Universidad San Sebastián, Valdivia, Chile. This study was initially approved by the Scientific Ethics Committee of Universidad San Sebastián (CEC-USS, No. 42-24, 11 April 2024). Subsequently, the Scientific Ethics Committee of the Los Ríos Health Service (No. 186, 12 June 2024) provided oversight of protocol amendments, which, in accordance with its procedures, were reported collectively upon study completion.

### 2.2. Recruitment

Participants were recruited through social media, posters, local radio announcements, and printed graphics displayed throughout the city. Informative sessions were held to present the study objectives, procedures, potential risks and benefits, and to address participants’ questions prior to obtaining written informed consent. Following their consent, participants attended a screening visit to verify their eligibility according to the inclusion and exclusion criteria. As part of the informed consent process, participants also signed an agreement authorizing the storage of their biological samples (serum, plasma, and peripheral blood mononuclear cells (PBMCs)) at Universidad San Sebastián (Valdivia, Chile) for future analyses.

### 2.3. Participants

Eligible participants were adults aged 18–65 years, with a body mass index (BMI) between 25.0 and 34.9 kg/m^2^, and fasting triglycerides between 100 and 215 mg/dL. Exclusion criteria included the following: BMI < 25 or >34.9 kg/m^2^; significant weight change (≥10% in the previous three months); fasting triglycerides > 215 mg/dL; blood pressure > 140/90 mmHg; presence of uncontrolled metabolic or chronic diseases (hypertension, dyslipidemia, type 2 diabetes), pregnancy/lactation, and allergies or intolerances to study products (lactose, wheat, seaweed, and sunflower oil); use of triglyceride-lowering drugs (e.g., Fibrates) or anticoagulants; recent blood donation (last 3 months); and excessive alcohol consumption (≥40 g daily).

### 2.4. Intervention

Each participant received both treatments in random order. The intervention consisted of one capsule per day containing 1 g of lipid extract from AlmegaPl^®^ 2515 (Qualitas Health Inc., Houston, TX, USA, operating under iwi life), an EPA-rich (250 mg/d EPA) oil derived from microalgae *Nannochloropsis* sp. against a placebo control treatment consisting of high-oleic sunflower oil (HOSO). HOSO was selected as the control oil because it is rich in monounsaturated fatty acids and contains very low levels of omega-6 linoleic acid, thereby minimizing potential confounding from n-6–derived metabolites while providing a metabolically neutral comparator, in omega-3 intervention effects.

Capsules were identical in size, weight, and packaging to ensure blinding. Adherence was assessed by counting the returned capsules at each crossover visit (visit 2 and visit 4). Participants were also asked to report any adverse events during each visit.

### 2.5. Aims

The primary aim of this trial is to determine the effectiveness of omega-3 fatty acids at nutritional doses in improving fasting serum triglyceride levels. Secondary aims include postprandial serum triglyceride levels (0, 2, 4, and 6 h); the expression of lipid metabolism genes, such as peroxisome proliferator-activated receptor alpha (PPARα) and sterol regulatory element-binding transcription factor 1 (SREBP1c isoform), in peripheral blood mononuclear cells (PBMCs) during fasting and 4 h after eating; plasma concentrations of low-grade inflammatory markers, including tumor necrosis factor alpha (TNFα) and interleukin-6 (IL-6), assessed at fasting and 4 h postprandial; and the atherogenic properties of very-low-density lipoproteins (VLDLs) in the fasting state, including their relative richness in triglycerides, remnant cholesterol, apolipoprotein C-I, and apolipoprotein C-III, in individuals from the Chilean population.

### 2.6. Study Visits

Participants attended five scheduled appointments, including one screening visit and four intervention visits corresponding to the two crossover phases. The screening visit was conducted to verify eligibility criteria and obtain informed consent. The intervention visits included the following: V1: baseline visit to initiate the first supplementation phase and conduct fasting and postprandial assessments; V2: end-of-phase visit to complete the first supplementation period and return unused capsules; V3: crossover baseline visit, conducted after a 10-week washout, to initiate the second supplementation phase and conduct fasting and postprandial assessments; and V4: final visit to complete the second supplementation period, return unused capsules, and perform final assessments.

#### Standardized High-Fat Meal Challenge

To assess the triglyceride postprandial response, a standardized high-fat meal challenge, locally adapted from a previously validated postprandial lipid test protocol [13], was included as a voluntary component of the study. The clinical research team prepared the meal, which consisted of three slices (90 g) of white bread (Ideal^®^, Ilford, UK), 60 g of full-fat butter (Colun^®^, La Unión, Chile), and 20 g of raspberry jam (Tía Lía^®^, Valdivia, Chile). According to the manufacturers’ nutrition data, this breakfast provided approximately 716 kcal, comprising 51.8 g of total fat, 53.1 g of carbohydrates and 9.4 g of protein, corresponding to 65% of total energy from fat, with 30% from carbohydrates, and 5% from protein (Supplementary Table S1). The breakfast was consumed in the university cafeteria within 20 min, with up to 1 L of water permitted throughout the day. Postprandial waiting periods (2, 4, and 6 h) were spent on university premises to ensure participants remained on site for subsequent blood collections. All participants who completed the postprandial challenge received a snack.

### 2.7. Study Procedures

Blood pressure was assessed after a 5 min seated rest using a calibrated automated sphygmomanometer (Bokang BK6022, Wenzhou Bokang Instruments Co., Ltd., Wenzhou, China) on the non-dominant arm. Three readings were obtained at 2 min intervals; the mean values of the second and third readings were recorded.

Blood samples were collected at baseline, and 2, 4, and 6 h after the high-fat meal challenge by trained personnel via standardized venipuncture, following CLSI guidelines [14]. A Vacutainer^®^ system (Becton Dickinson, Franklin Lakes, NJ, USA) with a 21G/23G butterfly needle was used. The antecubital area was disinfected with 70% alcohol for ≥30 s before puncture, and tubes were filled in the recommended order: serum separator (BD Vacutainer^®^ SST™, no. 367983), EDTA (BD Vacutainer^®^ K2EDTA, cat. no. 367861), and CPT™ (BD Vacutainer^®^ Cell Preparation Tube, cat. no. 362753). Serum tubes were centrifuged 30 min post-collection (1500× *g* for 15 min); EDTA tubes were centrifuged within 1 h (1500× *g* for 20 min); and CPT tubes were centrifuged within 2 h for peripheral blood mononuclear cell isolation using a centrifuge with a swinging-bucket rotor. All aliquots (0.5 mL) were stored at −80 °C until analysis.

Body composition (lean mass (kg), fat percentage, and basal metabolic rate (kcal/day)) and weight were measured using a bioelectrical impedance analyzer (Rice Lake X-Contact 350, Rice Lake Weighing Systems, Rice Lake, WI, USA) while participants were barefoot and wearing light clothing; 0.8 kg was subtracted for clothing weight. Height was measured with a stadiometer (SECA 213) to the nearest 1 mm, with participants in the Frankfurt plane, without shoes or headgear.

As part of the Case Report Form (CRF) [15], Questionnaires quantified the Food Consumption Trends Survey, dietary recall, medical history, mealtime habits (chrononutrion), and physical activity levels through the International Physical Activity Questionnaire (IPAQ), which is a validated questionnaire quantifying walking, moderate, and vigorous activities in MET-minutes/week [16]. Sedentary behavior was evaluated through daily sitting time. Additional lifestyle information, including smoking status (yes/no), and dietary habits (food frequency questionnaire), where a nationally adapted instrument was used [17], were collected via structured interviews conducted by trained staff. The Quantified Food Consumption Trends Survey was administered only to participants included in the study, to characterize their baseline dietary patterns. No dietary data was collected during the screening visit.

#### Harms

Potential risks associated with participation in the RIO-Study are considered minimal. Blood sampling may cause mild discomfort, pain at the puncture site, or small hematomas, and rarely lead to infection or an allergic reaction to materials. The standardized high-fat test meal may produce transient gastrointestinal discomfort such as bloating, nausea, or indigestion. Daily supplementation with omega-3 (AlmegaPl^®^) or high-oleic sunflower oil (HOSO) may lead to mild gastrointestinal disturbances (nausea, diarrhea, stomach discomfort) and, in rare cases, allergic reactions in individuals with hypersensitivity to algae, sunflower, or other capsule ingredients. Interactions with anticoagulant medication are recognized, and individuals using such drugs are excluded from participation. Both interventions are manufactured under quality standards to minimize risks of contamination.

Adverse events will be systematically assessed at each study visit through direct questioning and spontaneous reporting by participants. All events will be classified by severity (mild, moderate, severe), relatedness to the intervention (unrelated, possibly related, probably related), and outcome (resolved, ongoing, serious adverse event). Any unexpected adverse event will be reported immediately to the principal investigator. The study team will monitor participants closely throughout the trial and provide appropriate care as needed.

### 2.8. Future Determinations

Several laboratory analyses are planned with the biological samples collected during the trial. Serum triglyceride concentrations will be determined using an enzymatic colorimetric assay (GPO/PAP method without glycerol blank) in fasting and postprandial samples (0, 2, 4, and 6 h). Total RNA will be extracted from peripheral blood mononuclear cells (PBMCs) to evaluate the expression of lipid metabolism-related targets such as *PPARA* and *SREBP1c*, using cDNA synthesis followed by quantitative real-time PCR (qPCR), with normalization to housekeeping genes and quantification by the ΔΔCt method. Inflammatory biomarkers (TNFα, IL-6) will be assessed in fasting and 4 h postprandial plasma samples through enzymatic assays and ELISA. Very-low-density lipoprotein (VLDL) particles will be isolated from fasting plasma by density gradient ultracentrifugation and analyzed for triglyceride richness, remnant cholesterol, and apolipoproteins C-I and C-III content in order to characterize their atherogenic properties. In addition, stored samples may be used for exploratory determinations of other lipid-related or inflammatory biomarkers not originally specified in the project proposal; such analyses will remain hypothesis-generating and will be reported in future manuscripts.

### 2.9. Sample Size and Randomization

The sample size was calculated to detect a 15% reduction in fasting triglycerides (SD 0.25 mmol/L), with α = 0.05, power ≥ 80%, and an anticipated dropout rate of 10%, resulting in a required total of 40 participants. Participants were randomly allocated to one of two treatment sequences (A followed by B, or B followed by A) using a computer-generated deterministic Wichmann–Hill-based algorithm. This approach ensured adequate randomization and strict allocation concealment, preventing any possibility of predicting the assignment.

### 2.10. Blinding

Both participants and the clinical research team were blinded to treatment allocation throughout the trial. The capsules containing omega-3 (oil extracted from 1 g of *Nannochloropsis* sp. microalga, providing 250 mg of eicosapentaenoic acid) and the control (high-oleic sunflower oil, HOSO) were identical in size, shape, color, weight, and packaging, and labeled only with the participant code and study phase. This ensured that neither participants nor the research team involved in recruitment, assessments, or sample collection could identify the intervention. The trial statistician was also blinded to capsule coding. Only the principal investigator had access to the randomization key, which was securely stored and not opened until all data were collected, the database was locked, and the statistical analysis performed.

### 2.11. Statistical Analyses

We presented continuous variables with normal distributions as means and standard deviations, continuous variables with non-normal distributions as medians and interquartile ranges (1st–3rd quartiles), and categorical variables as proportions. Differences between participants in different groups (women/men) were assessed using *t*-tests for normally distributed continuous variables, Mann–Whitney U tests for non-normally distributed continuous variables, and chi-squared tests for categorical variables. Associations among continuous variables were studied using Spearman’s rank correlations adjusted for multiple comparisons using the Bonferroni correction. For the crossover analyses, a linear mixed-effects model (LMM) was used to evaluate treatment effects, including fixed effects for treatment period and sequence, and a random intercept for participant to account for within-subject variability. Baseline triglyceride values at the beginning of each period were included as covariates. Potential carryover effects were examined through the sequence term and further tested by including a first-order carryover variable in sensitivity analyses If statistically significant carryover is observed (*p* < 0.05), a period-1-only sensitivity analysis will be reported. The protocol already incorporates a 10-week washout period, designed to minimize residual EPA incorporation. Analyses were conducted in R (version 4.3.1).

## 3. Results

**Figure 2:**
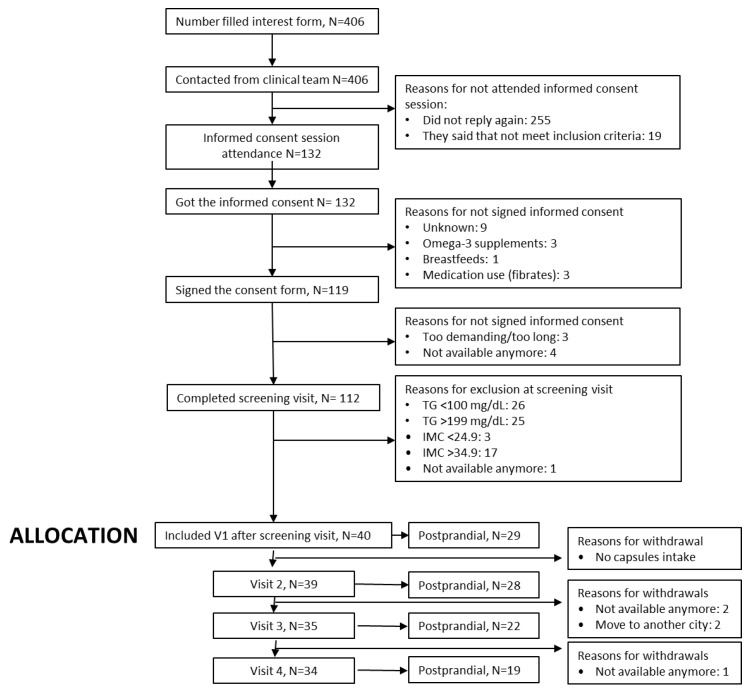
CONSORT flow diagram of the RIO-Study participant recruitment, allocation, follow-up, and analysis.

The CONSORT flow diagram (Figure 2) summarizes participant progression through the RIO-Study, from initial registration of interest to the completion of the follow-up phase. A total of 406 individuals initially expressed interest in participating in the trial, of whom 132 attended the in-person informed consent session. A total of 119 individuals provided signed informed consent. Screening assessments were performed with 112 participants, resulting in the enrolment of 40 eligible individuals who met all inclusion criteria and proceeded to Visit 1.

Baseline analysis of RIO-Study participants revealed a cardiometabolic risk profile characterized by excess adiposity, moderate dyslipidemia, and low physical activity in a considerable proportion of the cohort. The mean age was 40.6 ± 11.6 years, with no significant differences between sexes. However, men exhibited greater waist circumference and waist-to-hip ratio (100 ± 10.8 vs. 91.6 ± 11.9 cm; *p* < 0.001), higher systolic blood pressure (130 ± 12.0 vs. 122 ± 13.4 mmHg; *p* = 0.001), and higher levels of vigorous physical activity (2.40 [0.00;9.00] vs. 0.00 [0.00;0.36] (METs·min/week); *p* < 0.001), whereas women presented a higher proportion of normal-weight individuals (0% vs. 13%; *p* < 0.001), higher body fat percentage (31.1 ± 5.30 vs. 39.7 ± 4.75%; *p* < 0.001), and less daily sitting time (8.10 ± 2.28 vs. 6.83 ± 2.27 h; *p* = 0.006) (Table 1).

Regarding lipid profile (Table 2), no significant sex differences were observed in Apo-B, total cholesterol, low-density lipoprotein cholesterol (LDL-C), or non-high-density lipoprotein cholesterol (HDL-C). Nevertheless, men had significantly higher triglyceride levels (168 ± 84.7 mg/dL vs. 122 ± 64.9 mg/dL, *p* = 0.004), VLDL-C concentrations (33.7 ± 17.2 mg/dL vs. 24.4 ± 13.0 mg/dL, *p* = 0.004), and atherogenic index values (4.28 ± 1.22 vs. 3.59 ± 0.92, *p* = 0.002) compared to women. Conversely, women exhibited higher HDL-C levels (56.6 ± 13.3 mg/dL vs. 46.9 ± 9.39 mg/dL, *p* < 0.001). These differences indicate a less favorable lipid phenotype in men, characterized by elevated triglyceride-rich lipoproteins and reduced HDL-C concentrations, potentially contributing to higher cardiometabolic risk.

**Figure 3:**
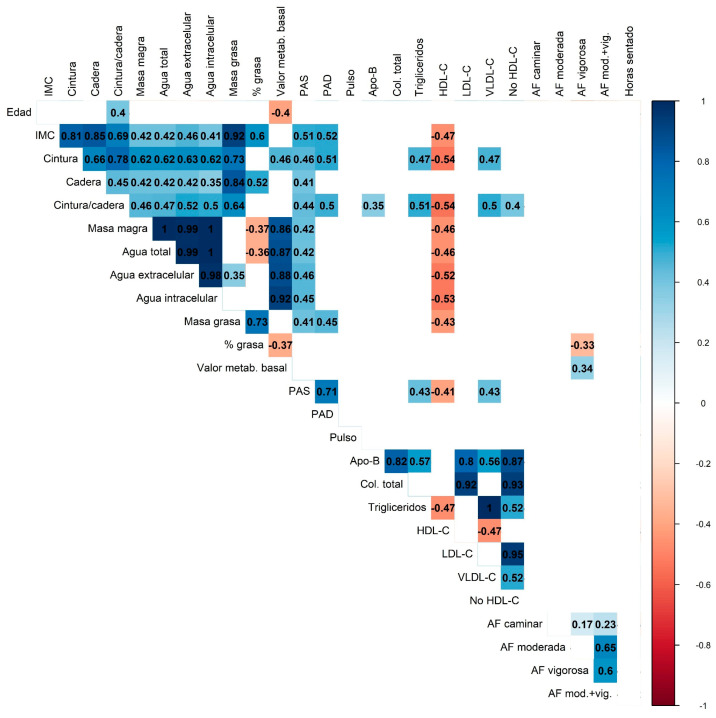
Correlation heatmap displaying the relationships between anthropometric, metabolic, and physical activity variables. The color scale represents the strength and direction of the correlations (blue for positive and red for negative correlations). Only statistically significant correlations are shown. Multiple comparisons were adjusted using the Bonferroni correction.

Correlation analysis (Figure 3) showed consistent patterns among anthropometric measures, body composition, and lipid profile parameters. Adiposity markers (BMI, waist circumference, fat mass) were strongly and positively correlated with each other and inversely correlated with HDL-C, while lean mass showed near-perfect correlations with total and compartmental body water. Apo-B, LDL-C, and total cholesterol formed a highly interrelated lipid cluster, with LDL-C and non-HDL-C displaying near-perfect collinearity. Moderate-to-vigorous physical activity was inversely associated with body fat percentage and triglyceride levels.

## 4. Discussion

The RIO-Study protocol is the first randomized, double-blind, placebo-controlled crossover clinical trial in Chile to examine the effects of nutritional-dose omega-3 fatty acids on fasting and postprandial lipid metabolism, inflammation, and molecular markers of lipid regulation. Its sequential design, including a standardized high-fat breakfast challenge with repeated postprandial sampling, allows detailed characterization of triglyceride kinetics, offering a dynamic assessment of lipid metabolism beyond fasting measures [18,19]. The integration of PBMC gene expression analyses (PPARα, SREBP1c) and inflammatory biomarkers (TNFα, IL-6) adds a mechanistic perspective, enabling exploration of pathways with the potential to mediate the cardiometabolic effects of omega-3 fatty acids [20,21]. Importantly, the focus on a Latin American population addresses a critical knowledge gap, as most prior studies have been conducted in North America and Europe [22], with a small amount of available data from Latin America [23]. Baseline characterization of the Valdivian population reveals a cardiometabolic risk profile marked by central obesity, atherogenic dyslipidemia, and low physical activity in a substantial proportion of participants, with no differences in age distribution between the sexes. Men exhibited greater waist circumference, waist-to-hip ratio, systolic blood pressure, and vigorous physical activity, whereas women showed higher body fat percentage and less daily sitting time. In lipid profiles, no sex differences were observed for Apo-B, total cholesterol, LDL-C, or non-HDL-C. However, men had higher triglycerides, VLDL-C, and atherogenic index values, while women displayed higher HDL-C values. These findings align with prior evidence of a more atherogenic lipid phenotype in men, likely influenced by hormonal factors, fat distribution, and lifestyle patterns [24,25]. Higher HDL-C values in women may confer partial protection, though this does not fully mitigate risks linked to central obesity and sedentary behavior [26].

Correlation analyses demonstrated strong positive associations between adiposity markers and triglycerides, and inverse associations between moderate-to-vigorous physical activity, body fat percentage, and triglycerides. Nearly half of participants failed to meet WHO physical activity recommendations, underscoring a significant behavioral and public health gap. These results reinforce the role of physical activity as a modifiable determinant of cardiometabolic risk [27,28].

The findings are consistent with national data from the Chilean National Health Survey 2016–2017, which reported overweight/obesity in 74.2% of adults and hypertriglyceridemia in 35.8% of adults [29]. The high prevalence of abdominal obesity, particularly in women, mirrors regional data linking this phenotype to insulin resistance and metabolic syndrome [30], enhancing the representativeness of the study population. These baseline results provide a robust foundation to assess the impact of nutritional-dose omega-3 supplementation, which has been shown in other cohorts to lower triglycerides, modulate lipid metabolism-related gene expression, and reduce inflammatory biomarkers [20,21,31]. The integration of molecular and functional endpoints in subsequent phases will clarify interindividual variability in response and support the development of personalized nutrition strategies in Latin America.

We anticipate several limitations of the RIO-Study. First, it is single-center and city-specific (Valdivia, southern Chile), which may restrict generalizability to other Latin American populations or individuals outside the eligibility window. Second, the modest sample size (*n* = 40), the 6-week intervention periods, and the use of nutritional rather than supra-nutritional doses of omega-3 fatty acids may result in limited changes for some secondary and mechanistic endpoints. The optional postprandial test may also introduce selection bias and missing data. Third, although the crossover design improves efficiency, residual carryover effects cannot be completely ruled out despite a 10-week washout. Fourth, the control oil (high-oleic sunflower oil) may exert minor metabolic effects and attenuate treatment contrasts when compared with an inert placebo; however, it was selected as the most biologically neutral option according to the previous literature. Fifth, background diet and physical activity were assessed mainly through questionnaires, which may lead to recall and misclassification bias. Finally, at the mechanistic level, gene expression and inflammatory profiling are restricted to selected targets (*PPARα*, *SREBP1c*, TNFα, IL-6) measured in fasting and 4 h postprandial states, while VLDL properties are assessed only in fasting conditions, which may not capture broader pathways or later postprandial dynamics.

## 5. Conclusions

The RIO-Study represents one of the first randomized, double-blind, placebo-controlled crossover trials in Chile to comprehensively evaluate the effects of nutritional-dose omega-3 fatty acids on fasting and postprandial lipid metabolism, inflammation, and molecular pathways regulating lipid homeostasis. The protocol integrates standardized high-fat challenge testing, repeated postprandial sampling, advanced biochemical profiling, and gene expression analysis in peripheral blood mononuclear cells. This multi-dimensional approach ensures high methodological rigor and provides a unique opportunity to generate mechanistic insights into omega-3 actions within a Latin American context, where clinical evidence regarding this issue remains scarce.

## Figures and Tables

**Table 1:** Baseline anthropometric, body composition, hemodynamic, and physical activity characteristics of participants by sex. Values are mean (SD) or median [Q1; Q3].

	SCR-All	SCR-Men	SCR-Women	*p*-Value
Age (years), mean (SD)	40.6 (11.6)	38.2 (11.8)	42.1 (11.2)	0.085
Body mass index (kg/m^2^), mean (SD)	30.8 (5.01)	31.0 (4.37)	30.7 (5.40)	0.712
BMI categories:				0.05
≤25 kg/m^2^ (*n*, %)	9 (8.04%)	0 (0.00%)	9 (13.0%)	
25.0–29.9 kg/m^2^ (*n*, %)	51 (45.5%)	22 (51.2%)	29 (42.0%)	
30.0–39.9 kg/m^2^ (*n*, %)	44 (39.3%)	19 (44.2%)	25 (36.2%)	
≥40 kg/m^2^ (*n*, %)	8 (7.14%)	2 (4.65%)	6 (8.70%)	
Waist circumference (cm)	95.0 (12.2)	100 (10.8)	91.6 (11.9)	<0.001
Abdominal obesity (*n*, %)	62 (55.4%)	19 (44.2%)	43 (62.3%)	0.093
Hip circumference (cm)	107 (8.75)	107 (7.34)	108 (9.55)	0.624
Waist-to-hip ratio	0.94 (0.08)	0.99 (0.08)	0.92 (0.07)	<0.001
Systolic blood pressure (mmHg), mean (SD)	125 (13.5)	130 (12.0)	122 (13.4)	0.001
Diastolic blood pressure (mmHg), mean (SD)	77.4 (9.78)	79.5 (9.93)	76.2 (9.53)	0.085
Heart rate (bpm), mean (SD)	69.6 (9.15)	66.3 (8.43)	71.6 (9.05)	0.002
Smokers (*n*, %)	35 (31.2%)	17 (39.5%)	18 (26.1%)	0.199
Physical activity: walking (METs·min/week), median [Q1; Q3]	231 [0.00; 462]	214 [0.00; 487]	240 [0.00; 462]	0.962
Moderate physical activity (METs·min/week), median [Q1; Q3]	240 [1.23; 520]	180 [0.00; 520]	300 [3.08; 520]	0.185
Vigorous physical activity (METs·min/week), median [Q1; Q3]	0.00 [0.00; 440]	2.40 [0.00; 9.00]	0.00 [0.00; 0.36]	<0.001
Sitting time (hours/day), mean (SD)	7.31 (2.35)	8.10 (2.28)	6.83 (2.27)	0.006
Lean mass (kg), mean (SD)	52.9 (10.8)	63.0 (8.99)	46.6 (5.84)	<0.001
Fat percentage (%), mean (SD)	36.4 (6.47)	31.1 (5.30)	39.7 (4.75)	<0.001
Basal metabolic rate (kcal/day), mean (SD)	1372 (224)	1579 (203)	1243 (113)	<0.001

SCR, screening; SD, standard deviation; BMI, body mass index; MET, metabolic equivalent task.

**Table 2:** Baseline lipid profile and apolipoproteins of the study population (*n* = 112).

	SCR-All	SCR-Men	SCR-Woman	*p*-Value
Apolipoprotein B (mg/dL), mean (SD)	81.7 (21.9)	85.0 (23.7)	79.7 (20.6)	0.233
Total cholesterol (mg/dL), mean (SD)	197 (39.4)	194 (39.7)	199 (39.4)	0.577
Triglycerides (mg/dL), mean (SD)	140 (76.2)	168 (84.7)	122 (64.9)	0.004
HDL cholesterol (mg/dL), mean (SD)	52.8 (12.8)	46.9 (9.39)	56.6 (13.3)	<0.001
LDL cholesterol (mg/dL), mean (SD)	119 (35.5)	119 (40.4)	118 (32.4)	0.876
VLDL cholesterol (mg/dL), mean (SD)	28.0 (15.3)	33.7 (17.2)	24.4 (13.0)	0.004
Non-HDL cholesterol (mg/dL), mean (SD)	144 (38.3)	147 (41.0)	142 (36.8)	0.543
Atherogenic index, mean (SD)	3.86 (1.09)	4.28 (1.22)	3.59 (0.92)	0.002

SCR, screening; HDL, high-density lipoprotein; LDL, low-density lipoprotein; VLDL, very-low-density lipoprotein.

## Data Availability

The data presented in this study are available on request from the corresponding author due to ethical restrictions given that personal data collected for this study.

## Supplementary Materials

The following supporting information can be downloaded at https://www.mdpi.com/article/10.3390/nu17213397/s1, Table S1: Nutritional composition of the standardized high-fat breakfast used in the RIO-Study.

## Abbreviations

CVD	Cardiovascular Disease
MetS	Metabolic Syndrome
EPA	Eicosapentaenoic Acid
DHA	Docosahexaenoic Acid
RIO-Study	Response to an Intervention with Omega-3 Study
BMI	Body Mass Index
PBMC	Peripheral Blood Mononuclear Cell
HOSO	High-Oleic Sunflower Oil
PPARα	Peroxisome Proliferator-Activated Receptor Alpha
SREBP1c	Sterol Regulatory Element-Binding Protein 1c
TNFα	Tumor Necrosis Factor Alpha
IL-6	Interleukin-6
VLDL	Very-Low-Density Lipoprotein
LDL-C	Low-Density Lipoprotein Cholesterol
HDL-C	High-Density Lipoprotein Cholesterol
VLDL-C	Very-Low-Density Lipoprotein Cholesterol
Apo-B	Apolipoprotein B
CRF	Case Report Form
IPAQ	International Physical Activity Questionnaire
MET	Metabolic Equivalent of Task
SD	Standard Deviation
CLSI	Clinical and Laboratory Standards Institute
ELISA	Enzyme-Linked Immunosorbent Assay
qPCR	Quantitative Polymerase Chain Reaction
ΔΔCt	Delta Delta Cycle Threshold (method for relative quantification)
ANID	Agencia Nacional de Investigación y Desarrollo
CEC-USS	Comité de Ética Científico—Universidad San Sebastián
NCT	ClinicalTrials.gov Identifier
