# Supplementary material for: Study Protocol and Baseline Cardiometabolic Characterization of the RIO-Study (Response to an Intervention with Omega-3): A Randomized, Double-Blind, Placebo-Controlled Crossover Trial on Lipid and Inflammatory Profiles in Overweight and Obese Adults with Hypertriglyceridemia in Valdivia, Chile
Source: Nutrients. 2025 Oct 29;17(21):3397. doi: 10.3390/nu17213397 (PMC12608147; doi:10.3390/nu17213397)
Supplement: Supplementary file 1 [file nutrients-17-03397-s001.zip › nutrients-3945756-supplementary.pdf]

Table S1. Nutritional composition of the standardized high-fat breakfast used in the RIO-Study.

Food item	Portion (g)	Energy (kcal)	Total fat (g)	*Saturated fat (g)*	*Monounsaturated (g)*	*Polyunsaturated (g)*	*Trans fat (g)*	Carbohydrates (g)	Protein (g)
**White bread (Ideal®)**	90	222	1.9	0.6	0.8	0.3	0	42.3	9.0
**Salted butter (Colún®)**	60	452	49.9	33.1	13.9	1.7	2.3	0.4	0.4
**Raspberry jam (Tía Lía®)**	20	42	0.02	0	0	0	0	10.4	0.04
**Total (Breakfast)**	—	716 kcal	51.8 g	33.7 g	14.7 g	2.0 g	2.3 g	53.1 g	9.4 g
